# Distinct immunological features of oropharyngeal cancer peritumoral tonsillar tissues from inflammatory tonsils and regional lymph nodes: A pilot study

**DOI:** 10.1371/journal.pone.0316102

**Published:** 2025-01-16

**Authors:** Naohiro Wakisaka, Makiko Moriyama-Kita, Satoru Kondo, Eiji Kobayashi, Takayoshi Ueno, Yosuke Nakanishi, Kazuhira Endo, Hisashi Sugimoto, Tomokazu Yoshizaki

**Affiliations:** 1 Department of Otorhinolaryngology, NHO Kanazawa Medical Center, Kanazawa, Ishikawa, Japan; 2 Division of Otorhinolaryngology and Head and Neck Surgery, Graduate School of Medical Science, Kanazawa University, Kanazawa, Ishikawa, Japan; University of Nebraska-Lincoln, UNITED STATES OF AMERICA

## Abstract

**Background:**

Cancer immune responses are generated in secondary lymphoid organs, such as the lymph nodes and tonsils. In the current study, transcriptional profiles of peritumoral tonsillar tissues (PTTs) from oropharyngeal cancers (OPCs) were assessed and compared with those of inflammatory tonsils and regional lymph nodes (rLNs).

**Methods:**

RNA samples of PTTs and rLNs from 13 OPCs, and 4 inflammatory tonsils were subjected to microarray analysis, and differentially expressed genes (DEGs) identified from 730 nCounter Panel immune-related genes. Gene Set enrichment Analysis (GSEA) was used for DEG profiling of PTTs and rLNs between lymph node metastasis-negative and metastasis-positive cases. The top 20 genes, as ranked by GSEA metric scores, were extracted and subjected to principal component analysis (PCA). The correlation of each patient’s PCA score with lymph node status was assessed by Receiver Operating Characteristics (ROC) analysis.

**Results:**

Comparing DEG analyses of PTTs with those of inflammatory tonsils and rLNs revealed 144 and 45 upregulated genes, respectively. ClueGO, a widely used Cytoscape plug-in, revealed activated pathways in PTTs, including lymphocyte proliferation (followed by T cell activation involved in the immune response) and positive regulation of leukocyte migration (followed by antimicrobial humoral immune response mediated by antimicrobial peptides) as the most significantly enriched immune system process functions in the gene ontology when comparing inflammatory tonsils and rLNs. The area under the ROC curves of PTTs and rLNs were 0.806 and 0.389, and were significant by DeLong’s test (p = 0.025).

**Conclusion:**

PTTs exhibit unique immunological features distinguishing them from inflammatory tonsils and rLNs. Gene expression analysis of PTTs is useful for investigating the mechanism of OPC lymphatic spread, even compared with analysis of rLNs.

## Introduction

Patient antitumor immune response significantly influences clinical outcome [1]. Thus, detailed tumor microenvironment characterization will translate to targeted therapeutic approaches and significant improvements in both clinical outcomes and quality of life following treatment [1]. Classically, immune responses to cancer are generated in secondary lymphoid organs (SLOs) [2,3]. During CD8^+^ T cell response initiation in SLOs, including regional lymph nodes (rLNs), naïve T cells are primed by dendritic cells (DCs) before migrating through the blood to the tumor [4,5]. However, in metastatic lymph nodes, cellular niches become more immunosuppressive, including elevated expression of inhibitory proteins towards DCs and regulatory T cells (Tregs) as well as more naïve and quiescent CD4^+^ T cells in head and neck cancers [5]. Thus, rLNs without metastasis are important in generating CD8^+^ T cell responses associated with anti-tumor immune responses.

Palatine tonsils, an SLO located in the oropharynx, are important for host defense against upper respiratory tract pathogens [6]. Our previous studies showed in oropharyngeal cancer (OPC) peritumoral tonsillar tissues (PTTs), the non-tumorous tonsillar tissues surrounding the primary tumor, the differential expression of many immune-related genes between lymph node metastasis-negative and metastasis-positive OPCs [7]. Thus, immunologic processes at PTTs of OPCs are likely crucial for effective immune responses by providing primary tumor sites with active immune cells. Additionally, mitochondria-related immunometabolic pathways are important for immunological regulation of lymphatic disease spread, for which the Toll-like receptor 4 (TLR4) cascade was most significant [8]. Thus, our previous findings highlight PTTs as important targets for investigation of immune mechanisms associated with OPC lymphatic dissemination. However, immunological differences between the PTTs, and rLNs or non-tumoral inflammatory tonsils are not yet elucidated. Furthermore, the clinical significance of investigating PTTs compared to metastasis-free rLNs, for elucidating immune mechanisms associated with the lymphatic spread of OPCs, still requires evaluation.

In this study, transcriptional profiles of inflammatory tonsils and rLNs without OPC metastasis were analyzed, and data compared with those of PTTs to further clarify PTT immunological features. PTTs and rLNs without metastasis were compared to evaluate their potential in predicting the lymphatic spread of OPCs. By examining these aspects, this study seeks to provide new insights into the immunological mechanisms underlying OPC lymphatic dissemination.

## Materials and methods

### Patient population

A total of 13 patients with oropharyngeal primary tumor radical resections and neck dissections performed for OPCs originating at the palatine tonsil were selected from the pathology files of Kanazawa University from 2017 to 2021 (Table 1). Patients were staged at diagnosis according to the eighth edition of TNM classification of malignant tumors of the Union Internationale Contre le Cancer [9]. The lymph node metastasis-positive patient classification includes all patients with lymph node metastasis at any stage of the clinical course. Of the 13 patients, 4 were lymph node metastasis-negative while the other 9 were metastasis-positive. The study protocol was approved by the Bioethics committee of Kanazawa University (number 2016–033). Written informed consent was obtained from all patients enrolled in this study.

**Table 1:** Clinical characteristics of the 13 patients with oropharyngeal cancer included in the study.

Factors		OPC patients (n = 13)
**Age**	**Median**	62 (43–84)
**Sex**	**Male**	12
	**Female**	1
**HPV**	**Positive**	11
	**Negative**	2
**Stage**	**I**	10
	**II**	1
	**III**	1
	**IVA**	1
**T classification**	**T1**	6
	**T2**	6
	**T3**	1
**Lymph Node Metastasis**	**Negative**	4
	**Positive**	9
**M classification**	**M0**	13

HPV, human papillomavirus; OPC, oropharyngeal cancer.

### Sample preparation

Samples from PTTs, non-tumorous tonsillar tissues surrounding the primary tumor, were prepared for each patient (S1 Fig). rLNs were analyzed for pan-cytokeratin expression to detect metastatic tumor cells, with only negatively stained lymph nodes selected for each patient. Inflammatory palatine tonsil samples were collected from 4 patients who underwent tonsillectomy from 2019 to 2020 for chronic tonsillitis. Finally, RNA was extracted from formalin-fixed paraffin-embedded (FFPE) tissue samples, 13 PTTs, 13 rLNs, and 4 inflammatory tonsils, using the RNeasy FFPE Kit (Qiagen, Tokyo, Japan).

### Microarray and computational analysis

The nCounter PanCancer Immune Profiling Panel (Nanostring Technologies, Seatle, WA) [10] was used to quantify 730 genes related to immune-cell profiling and identify differentially expressed genes (DEGs). The criteria for selecting DEGs was an adjusted *P* < 0.05. Volcano plot analyses for the 730 genes were performed using R packages, “ggplot2”, “ggrepel”, and “dplyr”. Data sets are available at the Gene Expression Omnibus database (https://www.ncbi.nlm.nih.gov/geo; accession number GSE244580). Data was analyzed on the 4th of August 2024. Authors had no access to information identifying individual participants during or after data collection.

Microarray molecular tests and computational analyses were performed using Cytoscape software version 3.10.3 (https://cytoscape.org) and its widely used plug-in, ClueGO. This tool enables the visualization of nonredundant biological terms within large DEG clusters in a group network and was used to interpret functionally grouped Gene Ontology (GO) pathway annotations related to the immune system process. A protein-protein interaction network of all DEGs was plotted using the Search Tool for the Retrieval of Interacting Genes/Proteins (STRING; https://string-db.org) to evaluate all protein-interaction associations. The protein-protein interaction networks were visualized using Cytoscape. To identify key high-level genes active in the protein-protein interaction network, hub gene analysis was performed using the NetworkAnalyzer tool in Cytoscape. The top 10 genes ranked by degree were considered hub genes. When multiple genes were ranked 10th, all were considered as hub genes.

### Gene set enrichment analysis and principal component analysis

Gene Set Enrichment Analysis software version 4.2.2 (GSEA; Broad Institute, Cambridge, MA, https://www.gsea-msigdb.org/gsea/index.jsp) was used for DEG profiling of PTTs and rLNs between metastasis-negative and metastasis-positive cases. The nCounter panel, with 730 immune-related genes, was used as the enrichment pathway. Then, data on the top 20 genes as ranked by GSEA metric score were extracted. Principal component analysis (PCA) was performed for the top 20 genes with principal component 1 extracted to serve as the gene signature score, then a PCA score for each patient was calculated. SPSS software version 28.0.2.0 (IBM, Armonk, NY) was used to perform PCA, and Receiver Operating Characteristic (ROC) analysis of PCA scores. The ROC curve and the Area Under the ROC Curve (AUC) were used to compare the effectiveness of identifying lymph node metastasis-negative and metastasis-positive cases in PTTs and rLNs. Differences between the AUCs of two curves were evaluated using DeLong’s test. *P* < 0.05 was regarded as statistically significant.

### Immunohistochemical staining

Consecutive 4-μm sections of PTTs were cut from each FFPE block. Immunohistochemical staining was performed as previously described [11,12]. The following antibodies were used as primary antibodies: rabbit CD40 polyclonal antibody (dilution 1:800) from abcam (Cambridge, UK); and rabbit CD40L/CD40LG antibody (dilution 1:100) from Boster Biological Technology (Pleasanton, CA). Sections were color-developed with substrate/chromogen diaminobenzidine (DAKO, Copenhagen, Denmark) with counterstaining by methyl green. Inflammatory tonsils were used as positive controls. Staining specificity was confirmed using nonimmune serum instead of the primary antibody as a negative control.

## Results and discussion

Inflammatory palatine tonsil tissue harbors various immune cell populations, including B cells, T cells, DCs, and macrophages, and forms an organized network of lymphoid follicles and interfollicular areas [6,13]. In the tonsil, B cells are abundant and actively undergo germinal center reactions leading to antibody production and affinity maturation. In the current study, to clarify immunological features between PTTs and inflammatory tonsils, the immunological transcriptional profile was assessed.

**Fig 1:**
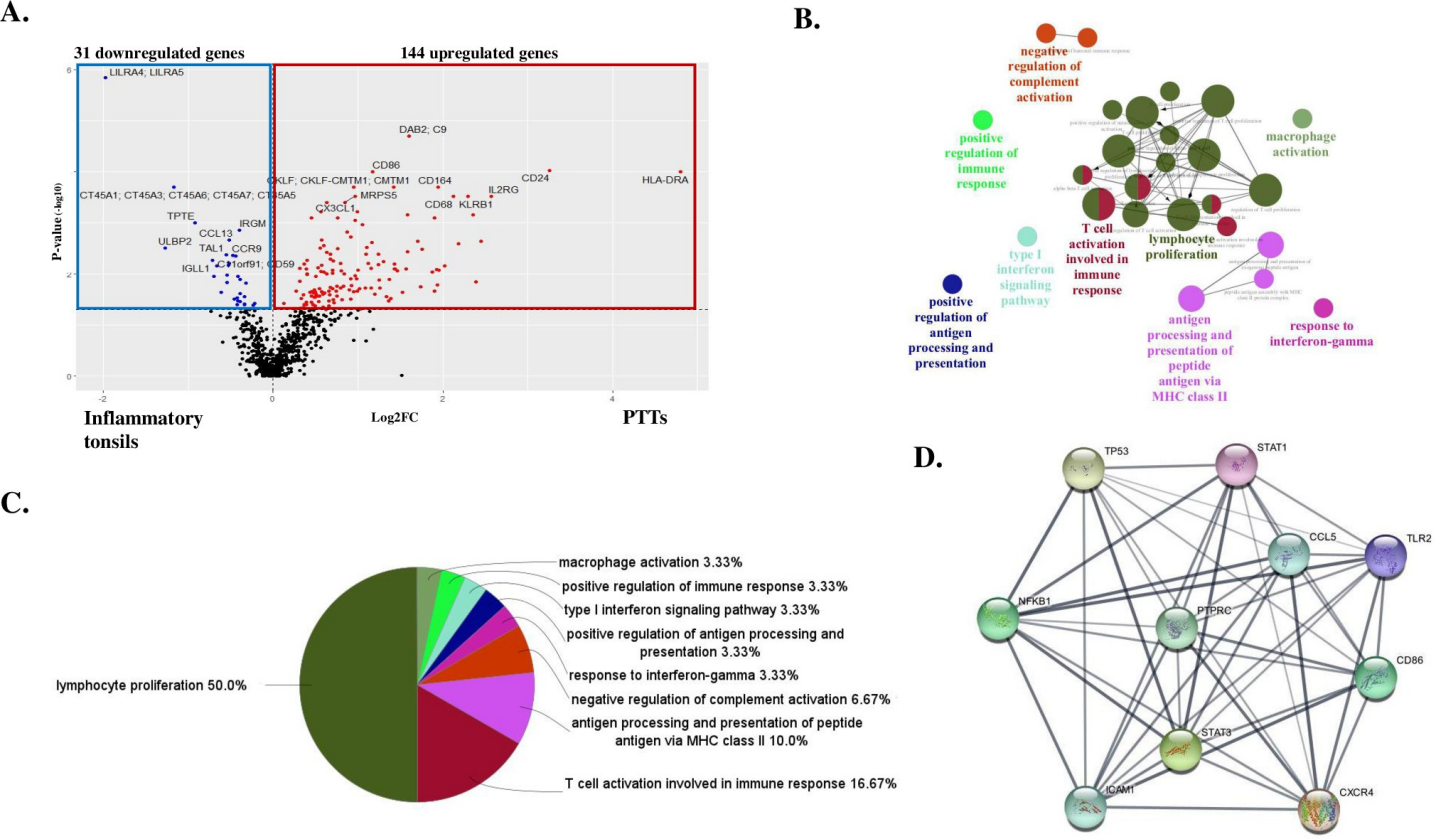
Gene ontology (GO) enrichment analysis of peritumoral tonsillar tissues (PTTs) compared with inflammatory tonsils. **A:** Volcano plot of differentially expressed genes between PTTs and inflammatory tonsils. **Red** and **blue dots** indicate up- and downregulated genes in PTTs compared with inflammatory tonsils. Statistical significance was defined as -log_10_ p-value > 1.3. Analyses of OPC PTTs revealed 144 upregulated and 31 downregulated immune-related genes compared with inflammatory tonsils. **B:** ClueGO analysis to identify immunological differences between PTTs and inflammatory tonsils. The interaction network of the GO terms in PTTs compared with inflammatory tonsils presented by the ClueGO plug-in for Cytoscape software version 3.10.3, used to decipher immune system process related variations in the GO. GO terms describing molecular interactions among targets are represented as nodes, while node size represents the term enrichment significance. The most significant term in each group is highlighted. **C:** Pie chart showing the percentage of each GO term in the PTTs compared with inflammatory tonsils. **D:** The top 10 hub genes are *PTPRC*, *CD86*, *CCL5*, *TLR2*, *STAT3*, *ICAM1*, *NFKB1*, *TP53*, *STAT1*, and *CXCR4*, suggested to play a central role in the upregulation observed in PTTs of oropharyngeal cancers compared to inflammatory tonsils.

RNA samples prepared from 13 PTTs in OPCs and from 4 inflammatory tonsils were analyzed to compare expression levels of 730 genes listed in the nCounter panel [10]. Of these, 175 genes were differentially expressed (144 upregulated and 31 downregulated in PTTs versus Inflammatory tonsils) (Fig 1A). For PTTs, the most significantly enriched in the GO immune system processes were lymphocyte proliferation, followed by T cell activation involved in the immune response, then antigen processing and presentation of peptide antigens via MHC class II (Fig 1B and 1C). These results suggest that compared with inflammatory tonsils, in which humoral immunity is predominant, tumor antigen-specific T cell activation as well as lymphocyte proliferation are observed in PTTs. Effective adaptive immune response against cancers usually occur in SLOs, wherein MHC molecule-peptide complexes are presented to CD4^+^ T and CD8^+^ T cells by mature DCs, requiring DC migration from the tumor site to the SLOs [2,3]. These steps allow lymphocyte proliferation and differentiation into effector T cells that migrate into the tumor and lead to tumor cell destruction [2,3]. Therefore, tumor antigen-specific CD4^+^ T cell activation via MHC class II at PTTs, a SLO, is significant for tumor antigen-specific adaptive immune response generation in OPCs. According to the ranking, the top 10 hub genes were *PTPRC*, *CD86*, *CCL5*, *TLR2*, *STAT3*, *ICAM1*, *NFKB1*, *TP53*, *STAT1*, and *CXCR4*, as visualized in Fig 1D. Hub genes, screened by protein-protein interaction network analysis, were involved in the activation of immunity reaction and inflammation pathways. The discovery of these specific immune-gene signatures in PTTs reveals essential details of tumor antigen-specific immunity in OPCs, and highlights the importance of immune mechanism clarification by further PTT investigation.

**Fig 2:**
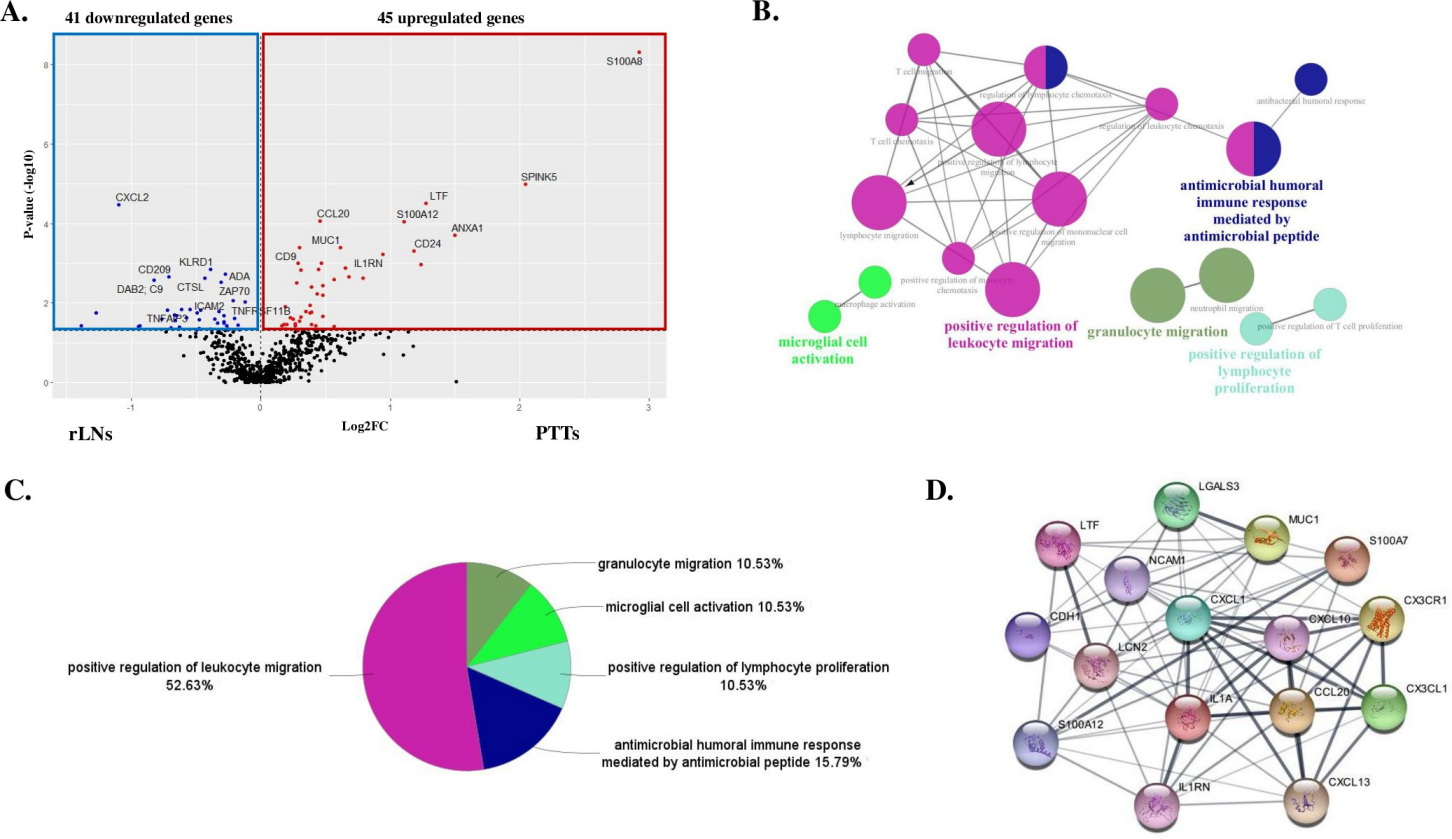
Gene ontology (GO) enrichment analysis of peritumoral tonsillar tissues (PTTs) compared with regional lymph nodes (rLNs). **A:** Volcano plot of differentially expressed genes between PTTs and rLNs. **Red** and **blue dots** indicate up- and downregulated genes in PTTs compared with rLNs. Statistical significance was defined as -log_10_ p-value > 1.3. Analyses of OPC PTTs revealed 45 upregulated and 41 downregulated immune-related genes compared with rLNs. **B:** ClueGO analysis to identify immunological differences between PTTs and rLNs. The interaction network of GO terms in PTTs compared with rLNs was generated using the ClueGO plug-in for Cytoscape software version 3.10.3, to analyze variations related to immune system processes in the GO. GO terms describing molecular interactions among targets are represented as nodes, while node size represents the term enrichment significance. The most significant term in each group is highlighted. **C:** Pie chart showing the percentage of each GO term in the PTTs compared with rLNs. **D:** The top 10 hub genes are *CXCL1*, *IL1A*, *CXCL10*, *LCN2*, *CCL20*, *IL1RN*, *CDH1*, *S100A12*, *LGALS3*, LTF, *NCAM1*, *CX3CL1*, *MUC1*, *CX3CR1*, *CXCL13*, and *S100A7*, are suggested to play key roles in the upregulation observed in PTTs of oropharyngeal cancers compared with rLNs.

Next, analyses of RNA samples from PTTs and rLNs from the corresponding 13 patients revealed 86 DEGs (45 upregulated and 41 downregulated in PTTs versus rLNs) (Fig 2A). The most significantly enriched pathways for PTTs compared with rLNs, were positive regulation of leukocyte migration, followed by antimicrobial humoral immune response mediated by antimicrobial peptide, then positive regulation of lymphocyte proliferation (Fig 2B and 2C). According to the ranking, the top 10 hub genes were *CXCL1*, *IL1A*, *CXCL10*, *LCN2*, *CCL20*, *IL1RN*, *CDH1*, *S100A12*, *LGALS3*, LTF, *NCAM1*, *CX3CL1*, *MUC1*, *CX3CR1*, *CXCL13*, and *S100A7*, as visualized in Fig 2D. These genes are involved in various biological processes, particularly inflammation, immune response, cell adhesion, and signaling. Among them, *LTF*, *S100A7*, and *S100A12* are associated with inflammatory responses towards antimicrobial activity [14,15]. The microbiome encompasses the entire ecological community including commensal, symbiotic, and pathogenic microorganisms inhabiting the human body in health and disease [16,17]. Human microbiome changes may promote tumor growth and contribute to carcinogenesis through mechanisms including chronic inflammatory response induction and anti-cancer immunity modulation [18,19]. Interestingly, OPC microbial signatures were distinct from those of oral squamous cell carcinoma [20]. Patients with OPCs had a gram-negative microbiome bias [16]. Furthermore, urogenital pathogens were associated with lymph node metastasis for patients with HPV-positive OPCs [16]. These data suggest that chronic inflammatory responses and/or modulation of anti-cancer immunity in PTTs, influenced by microbial infection from the resident flora, showed characteristic immune-gene signatures. These signatures are advantageous for the migration and/or influx of immune cells into PTTs followed by proliferation compared with rLNs.

Therefore, the PTT immune environment greatly differs from that of inflammatory tonsils and rLNs. An immunological feature of PTT is the activity of tumor antigen-specific T cell proliferation and response and immune cells migration, in part due to the difference of microbial signatures, supporting PTT as the tumor site source of activated tumor-specific immune cell [6,7].

**Fig 3:**
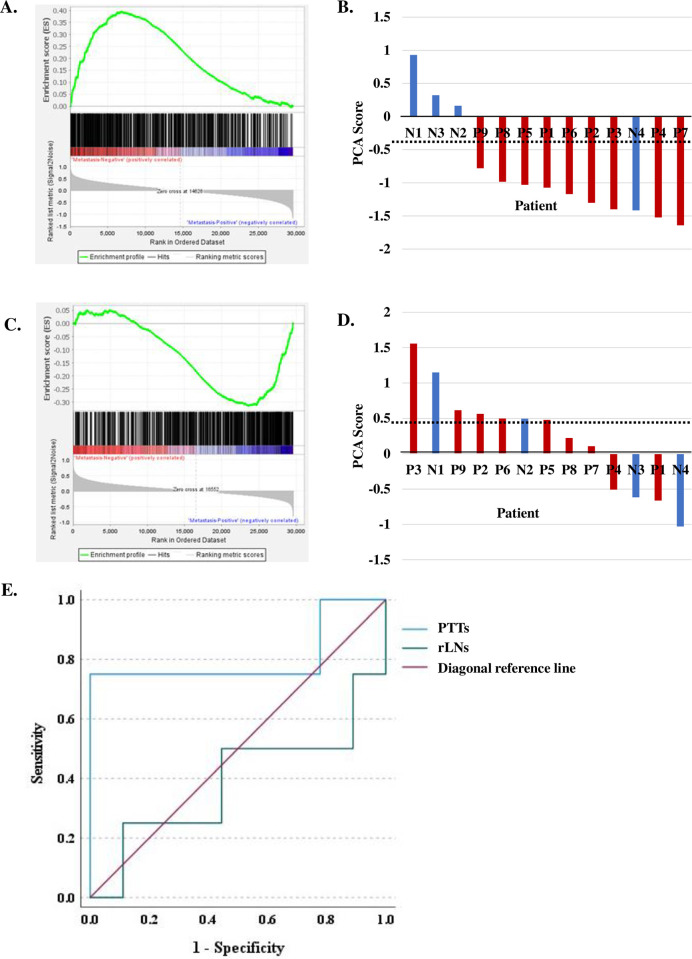
Gene Set Enrichment Analysis (GSEA) and principal component analysis (PCA) score. **A** and **C:** GSEA evaluations of 730 immune-related genes of the nCounter panel [10] between metastasis-negative and metastasis-positive cases in peritumoral tonsillar tissues (PTTs) **(A)** and regional lymph nodes (rLNs) **(C)**. Then, the top 20 genes ranked by GSEA metric score were extracted (Table 2). **B** and **D:** Relationships between patients’ lymph node status and PCA scores from Principal Component 1 in PTTs **(B)** and rLNS **(D)**. Dashed lines indicate PCA score thresholds for lymph node positivity, - 0.3081 for PTTs **(B)** and 0.4864 for rLNs **(D)**. Blue bars (N1 to N4) and red bars (P1 to P9) indicate lymph node metastasis-negative and metastasis-positive patients. **B.** According to the Receiver Operating Characteristic (ROC) analysis, 13 PTTs were segregated into two groups by PCA score: < - 0.3081 versus > - 0.3081 (sensitivity, 1.00; specificity, 0.75; accuracy 0.923). **D.** 13 rLNs were also segregated into two groups by PCA score: < 0.4864 versus > 0.4864 (sensitivity, 0.44; specificity, 0.50; accuracy 0.462). **E.** Area under the ROC curves for identifying lymph node metastasis-negative and metastasis-positive cases in PTTs and rLNs. Areas under the ROC curves are 0.806 and 0.389 for PTTs and rLNs. DeLong’s test for the two correlated curves showed a significant difference (p = 0.025).

GSEA from the nCounter panel [10] indicates that abundant immune-related genes from PTTs participate in lymph node metastasis suppression in OPCs (Fig 3A). The top 20 genes as ranked by GSEA metric score were extracted and applied to PCA. Three principal components were detected (Table 2), with the first principal component (approximately 82.748% variance) used to represent a patient’s PCA score. According to the ROC analysis, 13 PTTs were segregated into two groups by PCA score: < - 0.3081 versus > - 0.3081. The relationship between patients’ lymph node status and PTT PCA score are visualized in Fig 3B (sensitivity, 1.00; specificity, 0.75; accuracy 0.923). Immune-related genes from rLNs were also analyzed with GSEA followed by PCA score calculation for each patient (Fig 3C) (approximately 71.279% variance for the first principal component (Table 2). According to the ROC analysis, 13 rLNs were also segregated into two groups by PCA score: < 0.4864 versus > 0.4864. The relationship between patients’ lymph node status and rLNs PCA score are visualized in Fig 3D (sensitivity, 0.44; specificity, 0.50; accuracy 0.462). DeLong’s test, comparing the two correlated curves (area under the ROC curves for PTTs and rLNs are 0.806 and 0.389), showed a significant difference (p = 0.025) (Fig 3E). These results indicate that for nodal status prediction analyses, PTT RNA samples outperform rLNs samples. Thus, analyses of PTTs are more appropriate than rLNs to investigate the mechanism of lymphatic spread in OPCs. There are two possible reasons: 1. the induction of chronic inflammation necessary to provoke antitumor immunity in PTTs in proximity to microbiota, and 2. rLNs do not receive direct lymphatic drainage from the primary tumor site (unlike sentinel lymph nodes) [11,21], lowering their chance to elicit an immune response against the primary tumor. Therefore, PTTs appear more suitable than rLNs for assessing antitumor immune responses (without requiring sentinel lymph node extraction).

**Table 2:** Top 20 genes by rank metric score of gene set enrichment analysis and principal components of peritumoral tonsillar tissues and regional lymph nodes.

PTTs				rLNs			
Genes	Principal Components			Genes	Principal Components		
	1	2	3		1	2	3
***IKBKB***	0.999	-0.017	-0.039	***NRP1***	0.999	0.007	-0.034
***ATG16L1***	0.999	-0.041	0.032	***IL6ST***	0.981	-0.096	-0.166
***CASP8***	0.993	0.113	-0.032	***TRAF6***	0.963	0.241	-0.119
***HLA-DQA1***	0.991	-0.046	0.125	***BLK***	0.961	0.055	0.272
***IL4R***	0.983	0.182	-0.022	***CD46***	0.951	-0.295	-0.092
***TAP1***	0.98	0.19	-0.058	***CD3D***	0.95	-0.172	-0.26
***IL11RA***	0.976	0.11	-0.188	***MAPK8***	0.943	0.334	-0.006
***USP9Y***	0.966	0.118	0.23	***LY96***	0.936	-0.347	0.051
***DOCK9***	0.955	-0.289	0.057	***C7***	0.933	0.31	0.18
***ITGAL***	0.949	0.124	0.29	***THBS1***	0.92	-0.328	-0.214
***RPS6***	0.938	0.146	0.314	***CD28***	0.916	0.394	0.078
***CD84***	0.934	-0.179	-0.309	***IFNAR1***	0.912	-0.206	-0.354
***THBS1***	0.929	-0.369	0.014	***CXCL14***	0.9	-0.431	0.061
***PRG2***	-0.915	0.307	0.261	***EGR1***	-0.882	0.078	0.465
***APP***	0.905	-0.297	-0.304	***CASP8***	0.788	0.611	-0.08
***NOD1***	0.85	0.505	-0.147	***KLRD1***	0.607	0.793	-0.06
***STAT2***	0.847	0.371	-0.38	***CTSG***	-0.058	0.721	0.69
***FOS***	0.789	-0.57	0.228	***CD86***	0.687	0.044	0.725
***ENG***	0.78	0.404	0.478	***IRAK2***	0.27	-0.637	0.722
***PLA2G1B***	-0.124	0.985	-0.117	***SMAD3***	0.597	-0.378	0.707

PTTs, peritumoral tonsillar tissues; rLNs, regional lymph nodes. Principle Component 1 variances for PTTs and rLNs are 82.748% and 71.279%.

**Fig 4:**
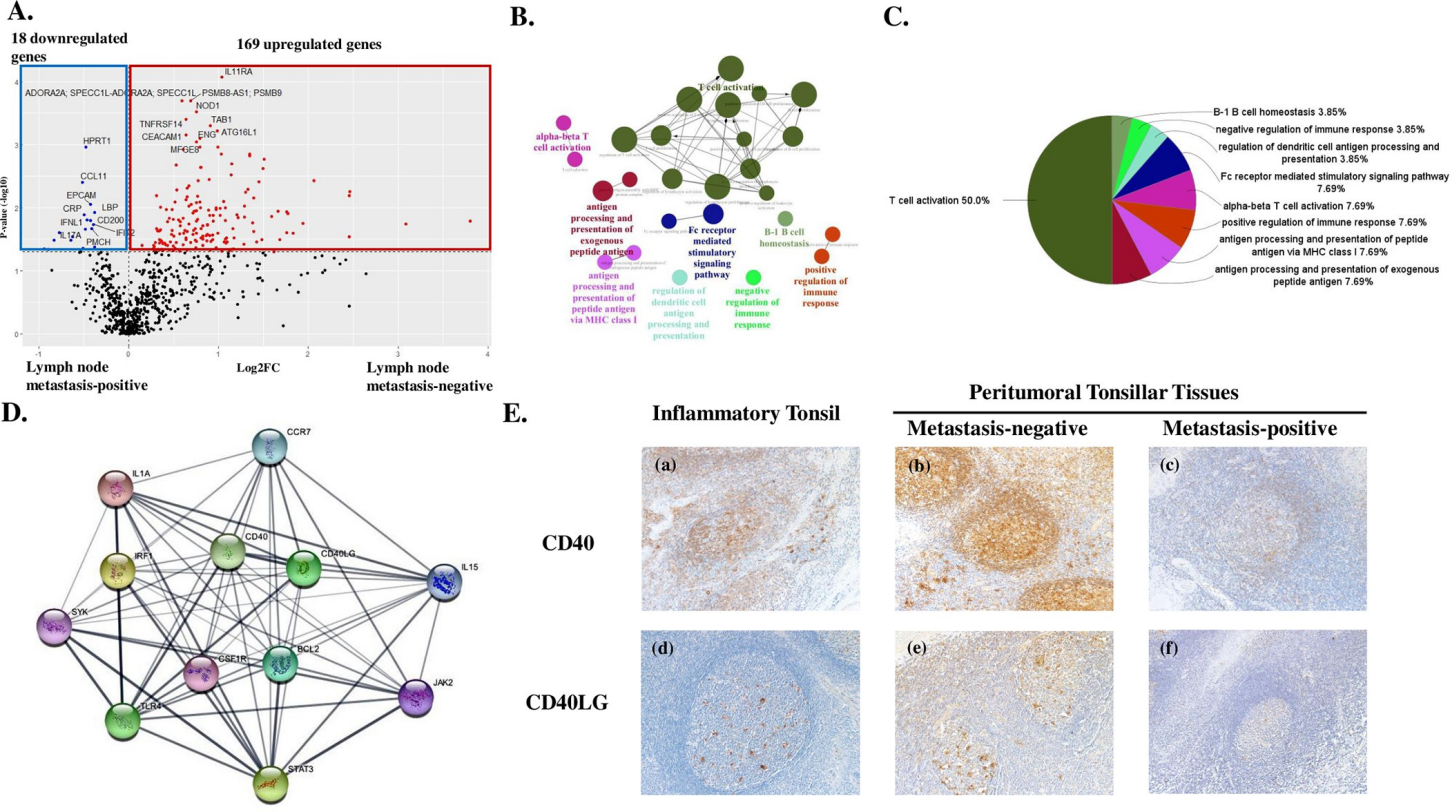
Gene ontology (GO) enrichment analysis of peritumoral tonsillar tissues (PTTs) from metastasis-negative cases compared with metastasis-positive counterparts. **A:** Volcano plot of differentially expressed genes between PTTs of metastasis-negative cases and those of metastasis-positive ones. **Red** and **blue dots** indicate up- and downregulated genes in PTTs of metastasis-negative and metastasis-positive cases. Statistical significance was defined as -log_10_ p-value > 1.3. Analyses of PTTs of metastasis-negative cases compared with metastasis-positive ones revealed 169 upregulated and 18 downregulated immune-related genes. **B:** ClueGO analysis to identify immunological differences between PTTs of metastasis-negative and metastasis-positive cases. The interaction network of the GO terms in PTTs of metastasis-negative compared with metastasis-positive cases presented by the ClueGO plug-in for Cytoscape software version 3.10.3, used to interpret immune system process related variations in the GO. GO terms describing molecular interactions among targets are represented as nodes, while node size represents the term enrichment significance. The most significant term in each group is highlighted. **C:** Pie chart showing the percentage of each GO term in the PTTs with metastasis compared with those without metastasis. **D:** The top 10 hub genes are *STAT3*, *TLR4*, *BCL2*, *CD40*, *IL1A*, *JAK2*, *CD40LG*, *IL15*, *SYK*, *CSF1R*, *CCR7*, and *IRF1*, suggested as central to upregulation in PTTs with metastasis of oropharyngeal cancers compared to those without metastasis. **E:** Representative photographs of CD40 and CD40LG immunostainings in inflammatory tonsils and PTTs, with and without lymph node metastasis of OPCs. Immunostaining of CD40 for (a) inflammatory tonsils, (b) PTTs in metastasis-negative cases, and (c) PTTs in metastasis-positive cases. Immunostaining of CD40LG for (d) inflammatory tonsils, (e) PTT in metastasis-negative case, and (f) PTTs in metastasis-positive cases. Magnification for each photograph is 100 ×.

To assess the activated lymphatic disease spread regulating pathway in PTTs of metastasis-negative OPCs, analyses of RNA samples from 13 PTTs involving 4 PTTs without metastasis and 9 with, revealed 187 DEGs (169 upregulated and 18 downregulated in metastasis-negative PTTs versus metastasis-positive) (Fig 4A). The most enriched pathways for metastasis-negative PTTs, compared with positive counterparts, were T cell activation, followed by antigen processing and presentation of exogenous peptide antigen, then antigen processing and presentation of peptide antigen via MHC class I (Fig 4B and 4C). According to the degree ranking, the top 10 hub genes were *STAT3*, *TLR4*, *BCL2*, *CD40*, *IL1A*, *JAK2*, *CD40LG*, *IL15*, *SYK*, *CSF1R*, *CCR7*, and *IRF1*, as visualized in Fig 4D. The TLR family consists of vital pattern recognition receptors responsible for innate immunity, and are core proteins involved in pathogen detection and immune response [22]. TLR4 is best known for the detection of lipopolysaccharide as a major pathogen-associated molecular pattern of gram-negative bacteria. TLR4 activation enhances both innate and adaptive immunity [23–25]. These data support the hypothesis of microbiota involvement in PTT immune activation to regulate OPC lymphatic spread. Microbiota analysis of OPC PTTs remains a target for future study. As confirmed in Fig 4E, CD40 and CD40LG are upregulated in PTTs of metastasis-negative OPCs. High levels of CD40 are usually found on B cells [26]. CD40LG expressed principally on activated T cells interacts with CD40, leading to a licensed state of APCs [27]. Licensed APCs upregulate costimulatory molecule expression, which further interacts with T cell activation mediators from the TNF receptor family, including CD27, 4-1BB and OX40 [28]. Importantly, CD40 stimulation enhances exogenous antigen cross-priming by APCs, resulting in efficient CD8^+^ T cell stimulation [29].

Although our clarification of immunological features of PTTs and prediction of nodal status in OPCs are encouraging, some study limitations exist. First, the analyzed cohorts were retrospective samples with limited numbers. The data from this pilot study requires further validation using commonly available data sets. However, no suitable datasets of PTTs were available for gene expression profile analysis to validate the data for the current study. Therefore, future validation of these results by multi-institutional studies is necessary. Second, rLN samples analyzed in the current study are from neck dissections. If sentinel lymph nodes were evaluated, their immunological features may differ from those of rLNs. Reevaluation using samples from sentinel lymph node biopsy specimens will address this. Third, the immuno-related pathway results were estimated from transcriptomic data, and require further verification through both multi-omics and *in vivo* analyses.

## Conclusion

Distinct immunological differences of PTTs from those of inflammatory tonsils and rLNs were observed. This demonstrated the importance of PTT sample gene expression analysis for investigating mechanisms of OPC lymphatic spread, even compared with rLNs analysis. Given these findings, medical researchers are advised to include PTT evaluations when investigating mechanisms of OPC lymphatic spread. This data provides the foundation for future multi-institutional large-scale validation studies.

## Supporting information

S1 Fig An example of peri-tumoral tonsillar tissue (PTT) macro-dissection from a lateral oropharyngectomy surgical specimen. Samples from PTTs, excluding the tumor area, were prepared for each patient, and were macro-dissected from whole formalin-fixed paraffin-embedded tissues on glass slide. ▲, tumor tissue; ★, PTT. The red frame indicates the PTT obtained by macro-dissection for microarray analysis.
(TIF)
