# Supplementary figures and images for: Distinct immunological features of oropharyngeal cancer peritumoral tonsillar tissues from inflammatory tonsils and regional lymph nodes: A pilot study
Source: PLoS One. 2025 Jan 16;20(1):e0316102. doi: 10.1371/journal.pone.0316102 (PMC11737673; doi:10.1371/journal.pone.0316102)

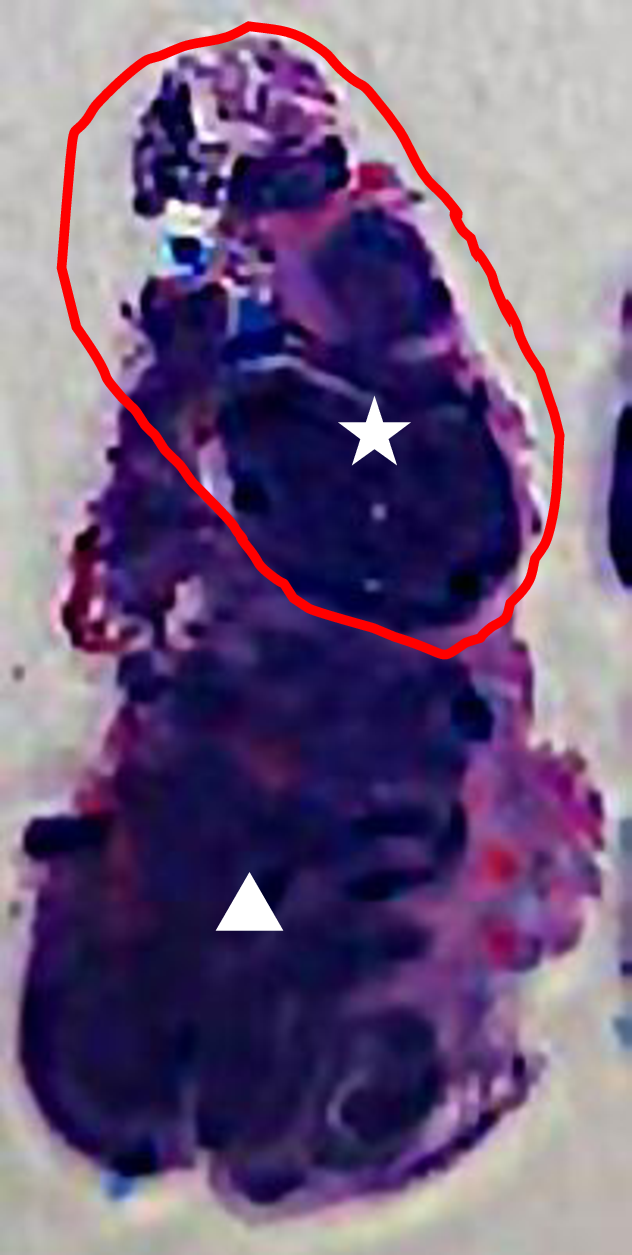

Supplement: S1 Fig — Samples from PTTs, excluding the tumor area, were prepared for each patient, and were macro-dissected from whole formalin-fixed paraffin-embedded tissues on glass slide. ▲, tumor tissue; ★, PTT. The red frame indicates the PTT obtained by macro-dissection for microarray analysis. (TIF) [file pone.0316102.s001.tif]
